# Mesoporous Silica Microparticle Tablets for Optimised Formulation and Overcoming Compressibility Challenges

**DOI:** 10.3389/bjbs.2025.14985

**Published:** 2025-11-17

**Authors:** Mohamad Anas Al Tahan, Craig Russell, Ali Al-Khattawi

**Affiliations:** 1 School of Pharmacy, College of Health and Life Sciences, Aston University, Birmingham, United Kingdom; 2 Aston Medical Research Institute, College of Health and Life Sciences, Aston University, Birmingham, United Kingdom

**Keywords:** mesoporous silica, microparticles, SYLOID, tablets, porosity

## Abstract

Tablets are the most commonly used dosage form due to their low manufacturing cost and ease of administration. Incorporating mesoporous silica microparticles offers enhanced control over drug release and bioavailability; however, formulation remains challenging due to poor compressibility and disintegration characteristics. This study explores dynamic formulation strategies to enable successful incorporation of SYLOID XDP 3150 (SYLOID) into oral tablet formulations. Tablets were prepared via direct compression using varying ratios of Avicel PH 102 (MCC: microcrystalline cellulose) and lactose monohydrate (25:75, 50:50, and 75:25) with SYLOID incorporated at 0%, 20%, and 40% (w/w). A 500 mg tablet mass was maintained throughout, and SYLOID alone was also compressed to assess baseline behaviour. Key tablet properties including porosity, tensile strength, friability, and disintegration time were evaluated. Direct compression of SYLOID alone failed due to poor compactability and particle fragmentation at 221.72 MPa. Increased Avicel content led to reduced porosity and enhanced tensile strength, while higher SYLOID levels increased porosity but compromised mechanical strength and friability. Disintegration was faster in lactose-rich formulations but delayed with increased SYLOID due to its hydrophobicity. Incorporating a superdisintegrant and binder enabled the final formulations to meet USP requirements for disintegration and friability. Overall, SYLOID was shown to significantly affect tablet architecture and performance, necessitating excipient support to overcome its inherent limitations. These findings support further evaluation of drug-loaded SYLOID tablets to assess their impact on drug release profiles and oral bioavailability.

## Introduction

Tablets are among the most widely utilised drug delivery systems (DDS) due to their simple and cost-effective production, particularly via direct compression (DC). However, despite its efficiency, DC is employed in fewer than 20% of pharmaceutical formulations, as both active pharmaceutical ingredients (APIs) and excipients must possess adequate compactability and compressibility for successful tabletting. As a result, different carriers and additives are frequently included in the powder blend to enhance tablet characteristics [1–3].

Silica has gained increasing prominence in pharmaceutical formulations owing to its biocompatibility, chemical inertness, and favourable safety profile [4–8]. It enhances compactability by increasing surface roughness, thereby improving interparticle bonding strength [9]. Nanoscale colloidal silica is widely used to improve powder flow by coating excipient surfaces, reducing interparticle friction, and minimising electrostatic interactions [10]. Silica microparticles also function effectively as glidants, helping to prevent sticking to tablet punches, while nanosilica contributes to improved tabletting by promoting particle rearrangement under pressure and reducing cohesion between particles [11–13].

The proportion of mesoporous silica in tablet formulations is a critical determinant of performance. Although small amounts (<1%) function well as glidants, higher concentrations may introduce cohesive forces that hinder powder flow and compromise compressibility [14]. However, the high porosity of mesoporous silica nanoparticles (MSNs) facilitates enhanced water uptake and volume expansion, which generate internal pressure to accelerate tablet disintegration. The low density and submicron size of MSNs ensure intimate contact with the dissolution medium, promoting rapid breakup of the tablet matrix [15, 16].

Compression forces also significantly influence the morphological properties of silica-based excipients. At elevated pressures, the porous architecture of silica can collapse, especially in the absence of plastic excipients such as microcrystalline cellulose (MCC) [17]. This mechanical stress may result in the fracturing of surface structures, reducing surface area and pore volume, key attributes for drug loading as they determine the available space for drug molecules to be adsorbed and encapsulated [18]. The nature of diluents used plays an essential role in mitigating these effects: MCC, being plastically deformable and insoluble, prolongs disintegration but protects silica pores, whereas lactose, which is water-soluble, supports faster disintegration due to its solubility and weak binding properties [19–21]. MCC also dissipates compression energy, preserving the structural integrity of porous carriers [18, 22].

Silica particle size is another factor influencing compactability and mechanical strength. Smaller silica particles tend to form more extensive interparticle bonds, enhancing tablet hardness [23, 24]. The specific type of silica also impacts final tablet properties: magnesium aluminosilicates generally produce harder tablets due to strong interparticle cohesion, while silanol-rich carriers such as SYLOID exhibit lower mechanical strength but benefit from greater disintegration potential due to improved wetting and pore channel formation [24–26].

This study aims to develop mesoporous silica microparticle tablets for oral delivery, focusing on the critical factors that influence their tabletting process and physical performance. “SYLOID XDP 3150 (referred to hereafter as SYLOID) is a commercially available grade of large-pore mesoporous silica, composed of irregularly shaped microparticles, known for its high surface area and suitability as a pharmaceutical excipient, was specifically selected for its high surface area, large particle size, and suitability for drug adsorption and controlled release applications. Its large particle size makes it distinct from the nanoscale silica materials commonly studied, offering potential advantages in handling and flow but also presenting unique compression challenges. While previous studies have broadly examined mesoporous silica in tablet formulations, the direct compression of mesoporous silica microparticles, particularly SYLOID XDP 3150 has not been systematically reported. This work addresses that gap by evaluating how compression forces affect the morphological integrity of SYLOID during tabletting, a factor often overlooked in silica-based tablet development. By systematically analysing the interactions between silica content, diluent ratio, and compaction pressure, this research aims to optimise tablet performance and provide a promising platform for the future development of drug-loaded formulations aimed at improving oral bioavailability.

## Materials and Methods

### Materials

Mesoporous silica microparticles SYLOID XDP 3150 were kindly provided by W.R. Grace and Co (Worms, Germany). Lactose monohydrate 316 fast flow was purchased from Foremost Farms (USA). Avicel PH-102 was kindly provided by FMC (Philadelphia, USA). Polyvinylpyrrolidone (PVP) average Mw 40.000 was purchased from Fisher Scientific (Belgium). Magnesium stearate (3.8%–5% Mg) was purchased from Acros Organics (Netherlands). Croscarmellose sodium was purchased from Spectrum Chemicals (UK).

### Methods

#### Morphological Properties

Imaging of the SYLOID particles was conducted using the Environmental Scanning Electron Microscope (SEM) mode of the ThermoFisher Scientific Quattro S microscope equipped with a field emission filament (FEG). The images were taken in low vacuum mode in variable pressure between 85 and 105 Pa, with an acceleration voltage of 3 kV and 3–3.5 spot size.

#### Particle Size Analysis Using Laser Diffraction

Particle size distribution of SYLOID, both in its original and compressed forms, was evaluated using laser diffraction (Sympatec GmbH, Germany). The system included a VIBRI vibratory feeder, a RODOS/L dry dispersion unit, and a HELOS/BR laser diffraction sensor. An R3 Fourier lens (focal length = 100 mm), with a detection range of 0.5–175 μm, was employed. Samples were dispersed at 1 bar pressure under vacuum (22 mbar), with a feed rate of 50% and optical concentration maintained at ≥1%. Data acquisition and particle size distribution analysis were performed using PAQXOS 5.0 software. All measurements were conducted in triplicate to ensure reproducibility.

#### Tablet Formulation: Influence of Powder Composition and Silica Content on Tablet Properties

To investigate the influence of powder composition on tablet performance (including assessments of hardness, disintegration time, and friability), blends were prepared using varying ratios of microcrystalline cellulose (Avicel PH102) and lactose monohydrate (25:75, 50:50, and 75:25, w/w). SYLOID was incorporated at concentrations of 0%, 20%, and 40% w/w relative to the total tablet weight. Following homogenisation, magnesium stearate (1% w/w) was added as a lubricant and mixed for an additional 5 min. Tablets (500 mg, 13 mm diameter, flat-faced) were compressed using a Power 8T automatic press (Specac, UK) equipped with a 13 mm evacuable pellet die. A compression pressure of 221.72 MPa was applied, and 30 tablets were produced per batch.

#### Tablet Formulation: Influence of Binders and Disintegrants on Friability and Disintegration

To address issues of excessive friability, polyvinylpyrrolidone (PVP) was added as a binder at 5% w/w to the SYLOID-diluent powder blend. The mixture was blended for 5 min, followed by the addition of magnesium stearate (1% w/w). Additionally, croscarmellose sodium (CMC-Na) was incorporated at 2% w/w as a superdisintegrant in selected formulations containing varying SYLOID concentrations and avicel:lactose ratios. Tablets were prepared using the same procedure as described above, with consistent tablet weight (500 mg) and geometry (13 mm flat-faced) across all formulations.

#### True Density

The true desnity was measured using helium pycnometry, the instrument was Multipycnometer from Quantachrome (Syosset, USA). One tablet with the weight of 0.5 g was put in micro sample cell and the true volume V_t_ was calculated based on Archimedes principle of fluid displacement, while the fluid is the helium gas that is able to penetrate the porous structure of the tablet to the pores of an 10^−10^ m of size, and is inert towards materials at normal temperatures [27]. The true volume V_t_ was calculated using the following equation:

$$V_{t}=V_{c}-V_{R}\;\left(P_{1}/P_{2}-1\right)$$

Where V_c_ is the sample cell volume (11.6029), V_R_ is the reference volume (6.2581), P_1_ is the atmospheric pressure, and P_2_ is the pressure change. V_T_ will be used to calculate the true density of the tablet according to the following equation:

True density=Tablet weight/True volume

The porosity of the tablet is calculated using this equation:

$$\text{Porosity}=\left[1\;–\;\left(\text{Bulk density}/\text{True density}\right)\right]*\;100$$

The bulk density is calculated through:

Bulk denisty=Tablet weight/Volume

The volume is the tablet can be calculated considering that is takes the shape of a cylinder using:

$$\text{Volume}=\pi \;*\;r^{2}\;*\;h$$

Where r is the radius, and h is the tablet’s thickness.

#### Tablet Hardness

Tablet hardness was determined using a Copley TBF 1000 Tablet Hardness Tester (Copley Scientific, UK), which was calibrated prior to analysis. For each formulation, three tablets were tested. The tensile strength (σ) was calculated using the following equation:

$$\sigma =\frac{2\;*\;hardness}{\pi \;*d*h\;}$$

Where σ is the tensile strength, d is the tablet diameter, and h is thickness of the tablet. All Measurements were done in triplicate and values reported as mean ± SD.

#### Tablet Disintegration

Tablet disintegration time was assessed using the DTG 100i Disintegration Tester (Copley Scientific, UK) equipped with a USP basket apparatus operating at 30 cycles/min. The medium was distilled water maintained at 37 °C ± 2 °C using a temperature control unit. One tablet was placed per basket, and three tablets per batch were tested. Disintegration time was recorded when all fragments passed through the mesh.

#### Tablet Friability

Tablet friability was evaluated using a Sotax F2 Friabilator (USP) from J. Engelsmann AG (Ludwigshafen, Germany). Twenty tablets were weighed before testing and rotated in the drum at 25 rpm for 4 min. Afterward, the tablets were carefully dusted to remove excess powder, and friability was calculated using the following equation:

$$\left({\text{weight}}_{\text{before}}-{\text{weight}}_{\text{afetr}}\right)/\;{\text{weight}}_{\text{before}}\;*100$$

#### Statistical Analysis

Statistical analyses of data were performed with SPSS 28 program by using one-way Analysis of Variance (ANOVA) coupled with a Tukey post-hoc test. All experiments were conducted in triplicate. All data was presented as mean ± SD, and P-value <0.05 is considered statistically significant.

## Results

### Tablet Compaction Trials Using SYLOID as a Standalone Excipient

Tablet compaction trials were conducted to evaluate the compressibility and mechanical integrity of SYLOID as a standalone excipient. Tablets were compressed at three different pressures: 73.91 MPa (equivalent to 1 ton force), 147.82 MPa (2 tons), and 221.72 MPa (3 tons). At the two lower pressures (73.91 MPa and 147.82 MPa), SYLOID powders failed to produce coherent tablets, indicating poor plastic deformation and bonding under compression. This failure can be attributed to SYLOID’s physicochemical characteristics, including its large irregular particle morphology, low bulk density, and inherent brittle nature. Such characteristics inhibit the formation of strong interparticulate bonds that are essential for tablet consolidation during compression.

At the highest applied pressure of 221.72 MPa, tablets visually appeared intact immediately after compression; however, these compacts disintegrated into a fine powder upon ejection from the die, demonstrating insufficient mechanical strength to withstand post-compression handling. Despite this fragility, the compacts allowed for preliminary morphological analysis of compressed SYLOID particles. The chosen pressure of 221.72 MPa served as the maximum achievable pressure to induce partial compaction for characterisation purposes, as lower pressures failed to produce tablets, and higher pressures risked damaging equipment or producing unacceptable tablet properties.

**FIGURE 1:**
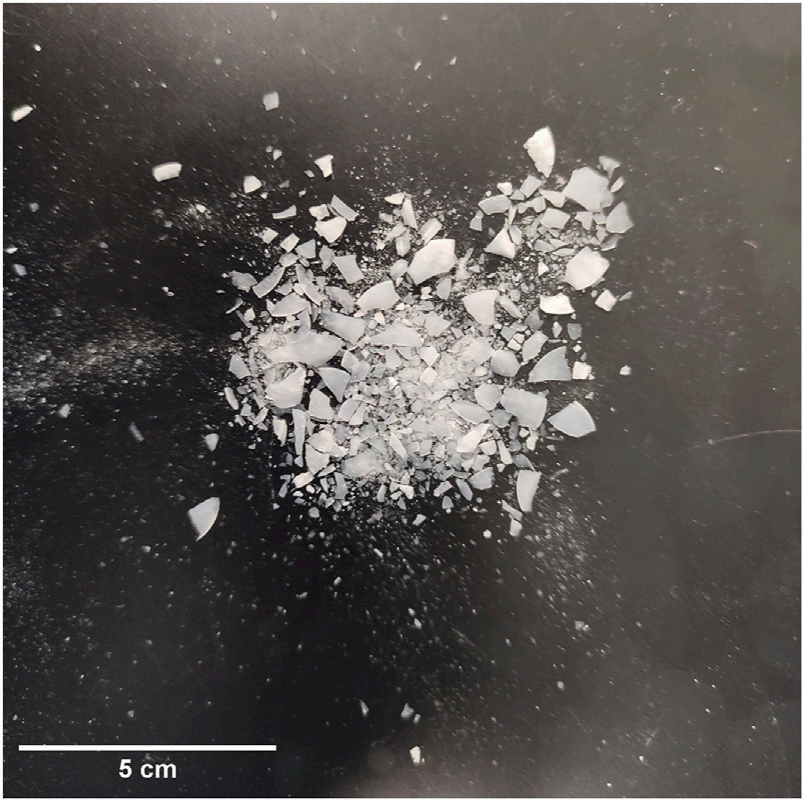
SYLOID tablet fragments after compaction at 221.72 MPa, showing large aggregates and fine powder formed upon tablet ejection.

Figure 1 illustrates the effect of compression on SYLOID particles. Under 221.72 MPa, SYLOID particles underwent significant fragmentation, compromising tablet integrity. As a mesoporous silica carrier, SYLOID is primarily composed of silicon dioxide (SiO_2_) with a porous internal network. Compression leads to structural squeezing, where the mesopores collapse under applied mechanical stress, adversely affecting mechanical strength and potentially releasing adsorbed or loaded compounds prematurely [28]. This structural deformation is more pronounced in SiO_2_-based carriers compared to aluminosilicate carriers, which generally show improved compressibility and tablet hardness due to their distinct chemical composition and microstructure [29].

Furthermore, SYLOID’s surface chemistry plays a role in its compressibility. Surface silanol (Si–OH) groups provide hydrophilicity but hinder the formation of strong interparticle bonds required for tablet robustness, limiting mechanical strength and hardness [26]. The poor plastic deformation capacity of SYLOID means that during compression, particles tend to fracture rather than deform plastically, resulting in increased brittleness. Particle size distribution analysis quantitatively confirmed these observations. The average particle size of compressed SYLOID was measured at 34.69 ± 8.13 μm, which was markedly smaller than the 117.3 ± 0.56 μm of uncompressed powder. This size reduction reflects extensive particle fragmentation during compression.

**FIGURE 2:**
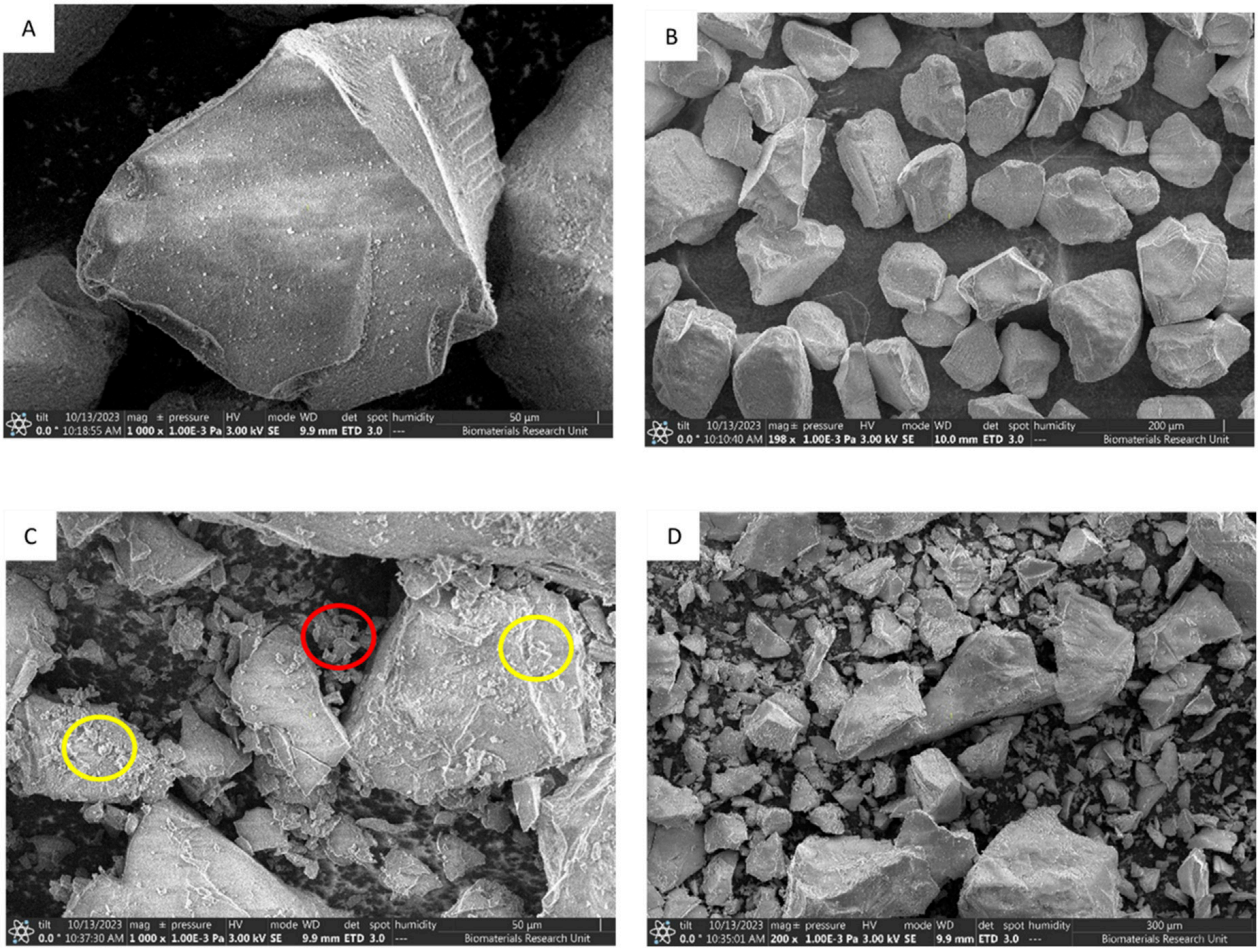
SEM imaging of SYLOID XDP 3150 at different magnifications showing the effects of compression force on the morphology of SYLOID particles before and after compressing at 3 Tons; SYLOID before compressing identifying the intact particle morphology **(A)**, powder population **(B)**, SYLOID particles after compression **(C)**, and after compression for the powder population **(D)**. The red circle represents the small fragments of SYLOID and the yellow present the cracks deformations on the carrier surface.

Scanning electron microscopy (SEM) images provided visual evidence of these changes (Figure 2). Uncompressed SYLOID particles (Figures 2A,B) appear as large, irregularly shaped particles with relatively smooth surfaces devoid of cracks or deformation. After compression, morphological changes are evident: numerous surface cracks (highlighted in yellow circles) and fractured silica fragments (red circles) are visible (Figure 2C). Detached debris was observed separately (Figure 2D), suggesting brittle fracture behavior. These findings are consistent with other studies demonstrating brittle fragmentation of mesoporous silica under compressive stress, leading to powder fines formation and reduction in particle integrity [24].

### Design of SYLOID-Based Tablets With Diluents

Given the poor compressibility of SYLOID alone, it was essential to investigate the effect of commonly used pharmaceutical excipients on tabletability. Microcrystalline cellulose (Avicel PH 102) and lactose monohydrate were selected as diluents due to their complementary physical properties and widespread use in direct compression formulations. Avicel PH 102 is known for its excellent plastic deformation and binding properties, whereas lactose is a brittle, water-soluble sugar excipient that facilitates disintegration.

Three different avicel:lactose ratios were prepared: 75:25, 50:50, and 25:75 (w/w). SYLOID was incorporated at two concentrations, 20% and 40% w/w of the total tablet weight, with magnesium stearate (1% w/w) added as a lubricant. All powders were thoroughly mixed to ensure homogeneity and then compressed into 500 ± 1 mg tablets using a flat-faced punch. This experimental design allowed assessment of how varying excipient proportions influence tablet porosity, disintegration, and mechanical strength, particularly with respect to SYLOID content. Table 1 summarises reports the values for the tests conducted on the tablets.

**TABLE 1:** Properties of tablets with varying avicel:lactose ratios and SYLOID concentrations, including disintegration, thickness, tensile strength, friability, and porosity. Tablets with 40% SYLOID and a 25:75 avicel:lactose ratio were excluded due to brittleness and instant disintegration upon ejection.

Formulation	Disintegration time (min)	Thickness (mm)	Tensile strength (MPa)	Friability (%)	Porosity (%)
avicel:lactose 25:75	1.92 ± 0.13	2.45 ± 0.01	15.85 ± 0.64	1.38 ± 0.23	21.93 ± 1.02
SYLOID 20% avicel:lactose 25:75	13.97 ± 0.21	2.6 ± 0.07	8.07 ± 0.33	5.46 ± 2.62	40.22 ± 1.54
SYLOID 40% avicel:lactose 25:75	Tablets were brittle and disintegrated instantly upon ejection				
avicel:lactose 50:50	7.15 ± 0.33	2.54 ± 0.02	27.24 ± 0.41	0.34 ± 0.18	23.08 ± 1.28
SYLOID 20% avicel:lactose 50:50	25.33 ± 0.7	2.73 ± 0.04	11.09 ± 0.64	2.79 ± 0.56	40.87 ± 1.2
SYLOID 40% avicel:lactose 50:50	More than 50 min	3.18 ± 0.03	8.99 ± 0.43	17.01 ± 1.45	52.28 ± 0.78
avicel:lactose 75: 25	28.08 ± 0.85	2.61 ± 0.01	35.36 ± 0.98	0.2 ± 0.02	17.32 ± 1.19
SYLOID 20% avicel:lactose 75:25	39.34 ± 1.18	3.06 ± 0.05	15.78 ± 0.11	1.3 ± 0.39	37.34 ± 0.96
SYLOID 40% avicel:lactose 75:25	More than 50 min	3.40 ± 0.01	13.27 ± 0.37	3.49 ± 0.15	57.73 ± 0.85

### Porosity Analysis of SYLOID-Containing Tablets

**FIGURE 3:**
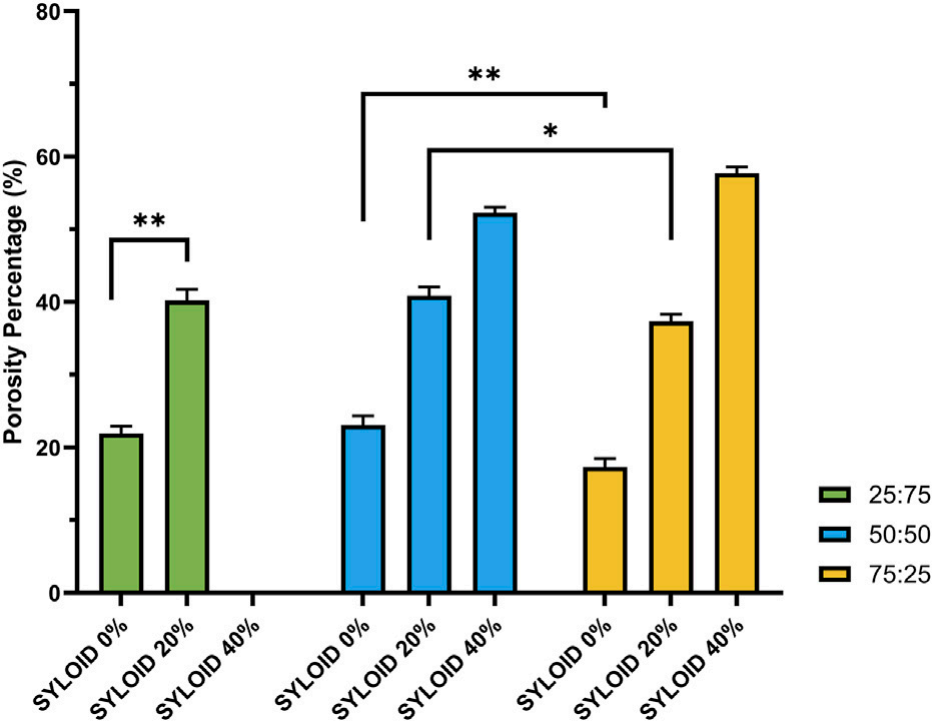
The effects of the diluent composition and SYLOID amount on the porosity of the tablets. The formulation containing 40% with (avicel:lactose ratio of 25:75) is not included as it disintegrated instantly upon ejection. Error bars representing standard deviation and *p ≤ 0.05 and **p ≤ 0.01.

Porosity is a critical parameter influencing tablet mechanical properties and dissolution. Figure 3 depicts the porosity values of the tablet formulations with varying diluent ratios and SYLOID loadings. A clear pattern emerges: increasing SYLOID content leads to increased tablet porosity across all formulations, consistent with SYLOID’s low density and porous nature.

Notably, tablets with 40% SYLOID at 25:75 avicel:lactose disintegrated immediately upon ejection, precluding accurate porosity measurement. These tablets were excluded due to inadequate mechanical strength. Tablets with 40% SYLOID and 75:25 avicel:lactose exhibited the highest porosity (57.73% ± 0.85%), significantly higher than SYLOID-free tablets at the same diluent ratio (17.32% ± 1.19%). This substantial increase indicates that SYLOID’s porous particles introduce voids within the tablet matrix, increasing overall tablet porosity. Generally, porosity decreased as Avicel content increased (p < 0.01). This trend reflects Avicel’s denser packing and stronger interparticle bonding compared to lactose, which inherently produces more porous compacts due to its brittle fracture mechanism [18, 21]. Microcrystalline cellulose’s fibrous structure and hydrogen bonding facilitate plastic deformation and close packing, reducing porosity [30, 31].

SYLOID’s contribution to porosity arises from its intrinsic high pore volume (1.7 cm^3^/g), which cannot be compressed or filled by other excipients [32]. As SYLOID concentration increased from 0% to 20%, tablet porosity rose significantly. For instance, 25:75 avicel:lactose tablets saw porosity increase from 21.93% ± 1.02% to 40.22% ± 1.54%, demonstrating SYLOID’s pronounced effect.

### Disintegration Time of SYLOID Tablets

**FIGURE 4:**
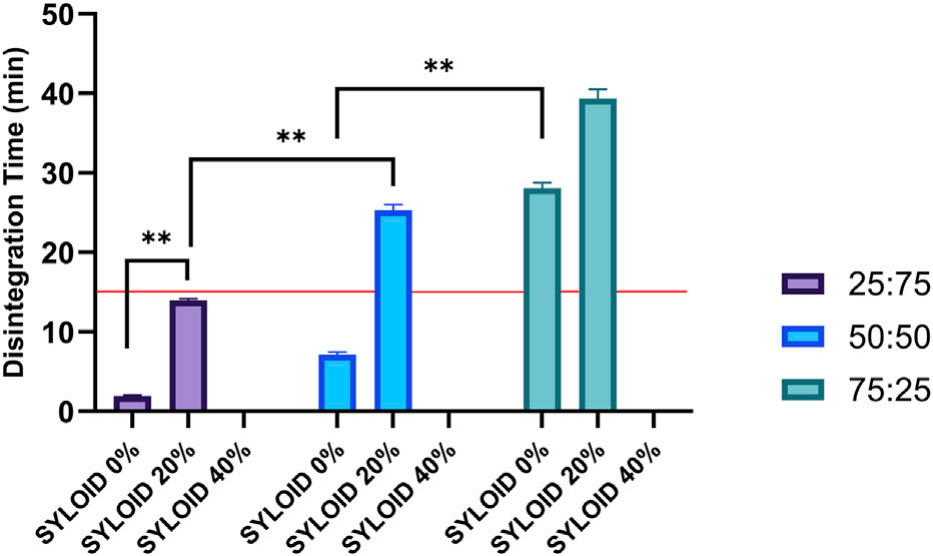
The effects of the diluent composition and SYLOID amount on the disintegration time of the tablets. The tablets with 40% SYLOID and made using both avicel:lactose ratios of 50:50 and 75:25 was not added as they did not disintegrate after 50 min. The formulation containing 40% with (avicel:lactose ratio of 25:75) is not included as it disintegrated instantly upon ejection. The redline represents 15 min disintegration time of the USP. Error bars representing standard deviation and **p ≤ 0.01.

Disintegration time is a key quality attribute impacting drug release kinetics and bioavailability. Table 1 summarises disintegration times, while Figure 4 presents the effect of excipient ratios and SYLOID content on disintegration. Tablets containing 40% SYLOID with 25:75 avicel:lactose disintegrated immediately upon ejection due to mechanical brittleness and lack of cohesion, thus excluded from detailed disintegration analysis. Similarly, 40% SYLOID tablets with 50:50 and 75:25 diluent ratios failed to disintegrate within 50 min, indicating excessively slow disintegration or potential capping.

Formulations with 20% and 40% SYLOID at 75:25 avicel:lactose ratios exhibited the longest disintegration times, exceeding USP specifications for uncoated tablets (disintegration ≤15 min) [33]. Highlighting limitations in achieving timely tablet breakup at high SYLOID and Avicel concentrations.

Increasing Avicel content significantly prolonged disintegration (p < 0.01). For example, in 20% SYLOID tablets, disintegration times increased from 13.98 ± 0.21 min at 25:75 ratio to 39.34 ± 1.18 min at 75:25 ratio, reflecting Avicel’s low swelling and water absorption capacity, resulting in slower disintegration [18, 21]. Similarly, increasing SYLOID concentration prolonged disintegration times across all ratios (p < 0.01). At 50:50 avicel:lactose, disintegration time increased from 7.15 ± 0.33 min without SYLOID to 25.33 ± 0.7 min at 20% SYLOID loading.

The observed behaviour is attributed to the distinct physical properties of the excipients. Lactose monohydrate is water-soluble and rapidly dissolves upon contact with aqueous media, enhancing water penetration and tablet breakup [19, 20]. Conversely, MCC is insoluble and exhibits poor swelling, creating a dense matrix that impedes water ingress, thereby extending disintegration [18, 21]. Additionally, lactose’s solubility prevents formation of impermeable layers, promoting faster disintegration [34]. SYLOID’s micro-sized particles (∼115 μm) differ substantially from conventional mesoporous silica nanoparticles (MSNs), which have much smaller particle sizes and higher surface areas. These features in MSNs facilitate rapid solvent absorption and tablet disintegration [15]. The larger SYLOID particles result in fewer particles per tablet and occupy a greater volume, reducing overall surface area available for solvent contact and thereby prolonging disintegration. Differences in disintegration among SYLOID-free tablets are consistent with porosity and excipient composition. Higher porosity tablets enable quicker solvent penetration, facilitating excipient dissolution and formation of voids, which accelerates disintegration [35].

**FIGURE 5:**
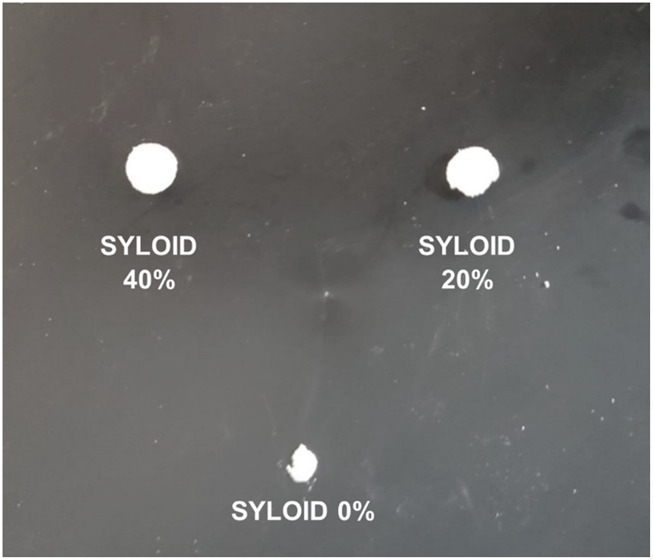
The disintegration and size decrease of tablets made with avicel:lactose 75:25 and different concentrations of SYLOID after leaving for 25 min.

Figure 5 visually demonstrates these trends, showing tablets with 75:25 avicel:lactose and varying SYLOID contents after 25 min in disintegration media. Tablets with 40% SYLOID showed minimal erosion, consistent with the longest disintegration times recorded. In contrast, 20% SYLOID tablets exhibited intermediate erosion and disintegration (∼39 min), and tablets without SYLOID disintegrated within 28.08 min.

### Effects of the Excipient Compositions and Silica Amount on Tensile Strength and Friability

**FIGURE 6:**
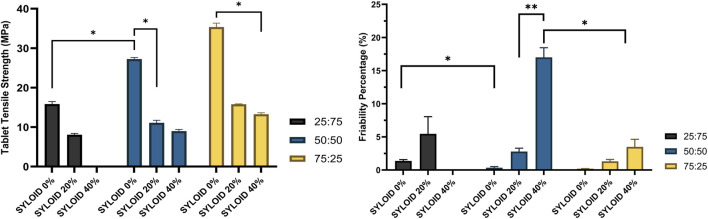
The effects of the diluent composition and SYLOID amount on the tensile strength (left) and friability (right) of the tablets. The formulation containing 40% with (avicel:lactose ratio of 25:75) is not included as it disintegrated instantly upon ejection. Error bars representing standard deviation and *p ≤ 0.05.

Figure 6 presents the tensile strength values (left) and friability percentages (right) for all tablet formulations studied. The formulation containing 40% w/w SYLOID with an avicel:lactose ratio of 25:75 was excluded because the tablets were highly brittle and disintegrated immediately upon ejection from the press, making tensile strength and friability measurements unfeasible.

The tensile strength values significantly increased (p < 0.05) with increasing Avicel content in the diluent mixture. Specifically, tensile strength rose from 15.85 ± 0.64 MPa at an avicel:lactose ratio of 25:75, to 27.24 ± 0.41 MPa at 50:50, and further to 35.36 ± 0.98 MPa at 75:25.

In contrast, an increase in SYLOID content caused a significant decrease in tensile strength in all formulations. For the formulation with avicel:lactose 75:25, tensile strength dropped markedly from 35.36 ± 0.98 MPa with 0% SYLOID to 13.27 ± 0.37 MPa at 40% SYLOID (p < 0.05). Similar trends were observed with the other ratios (25:75 and 50:50).

Regarding friability (Figure 6, right), the highest friability was seen in the formulation containing 40% SYLOID and avicel:lactose 50:50 (17.01% ± 1.45%), while the lowest was in the formulation with only avicel:lactose 75:25 (0.2% ± 0.02%). Friability significantly decreased (p < 0.05) from 1.38% ± 0.23% to 0.34% ± 0.18% when the avicel:lactose ratio increased from 25:75 to 50:50 in formulations without SYLOID. Conversely, friability significantly increased (p < 0.01) from 2.79% ± 0.56% to 17.01% ± 1.45% with an increase in SYLOID content from 20% to 40% in formulations with avicel:lactose 50:50.

### Effects of the Excipient Compositions and Silica Amount on Tablet Thickness

**FIGURE 7:**
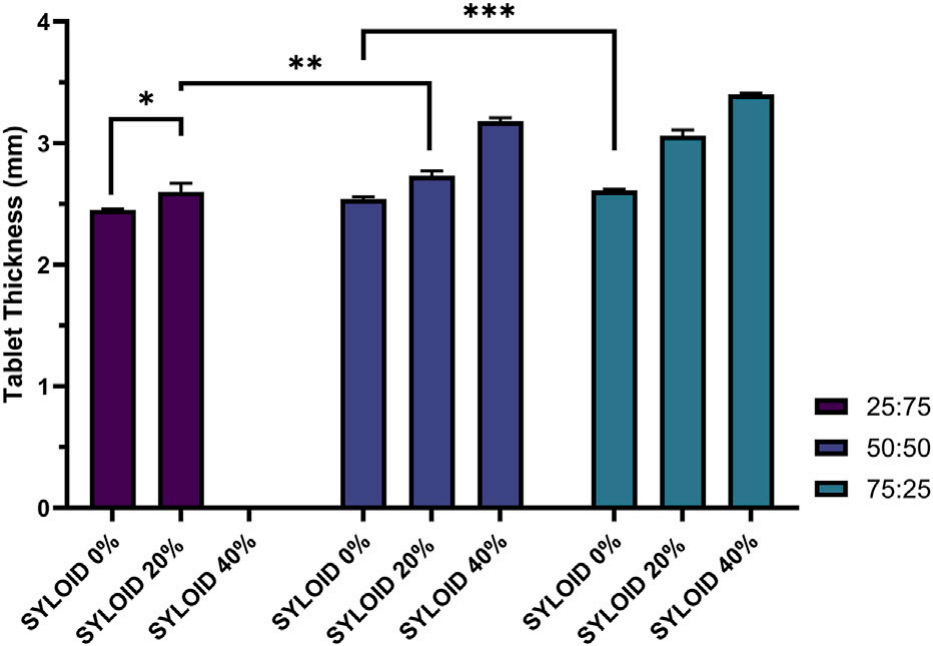
The effects of the diluent composition and SYLOID amount on the thickness of the tablets. The formulation containing 40% with (avicel:lactose ratio of 25:75) is not included as it disintegrated instantly upon ejection. Error bars representing standard deviation *p ≤ 0.05, **p ≤ 0.01, and ***p ≤ 0.001.

Tablet thickness data are summarised in Figure 7. The formulation with avicel:lactose 25:75% and 40% SYLOID was again excluded due to immediate disintegration post-ejection. Tablet thickness increased significantly with both higher Avicel content and increased SYLOID percentage. The thickest tablets (3.4 ± 0.01 mm) were observed in the 40% SYLOID and avicel:lactose 75:25 formulation. Increasing the Avicel ratio from 25% to 50% resulted in a significant thickness increase from 2.6 ± 0.07 mm to 2.73 ± 0.04 mm (p < 0.01). Similarly, raising SYLOID content in formulations correlated with increased tablet thickness.

### Effects of the Binder and Superdisintegrant on Tablet Properties

Table 2 shows the effects of incorporating polyvinylpyrrolidone (PVP) at 5% w/w and croscarmellose sodium (CMC-Na) at 2% w/w into the tablet formulation containing 20% SYLOID and avicel:lactose 75:25. Adding PVP significantly reduced friability from 1.3% ± 0.39% to 0.72% ± 0.02% (p < 0.05). The disintegration time remained above 15 min with PVP alone. The addition of CMC-Na as a superdisintegrant dramatically reduced disintegration time to 0.68 ± 0.01 min. The combined presence of PVP and CMC-Na resulted in tablets that met USP requirements for friability (<1%) and disintegration time (<15 min).

**TABLE 2:** Tablet properties after the addition of the binder (PVP) at a concentration of 5% w/w, and the superdisintigrant (croscarmellose Na) at 2% w/w.

Formulation	Disintegration time (min)	Thickness (mm)	Tensile strength (MPa)	Friability (%)	Porosity (%)
SYLOID 20% avi:lact 75:25	28.08 ± 0.85	3 ± 0.08	16.1 ± 0.11	1.3 ± 0.39	37.34 ± 0.96
SYLOID 20% avicel:lactose 75:25 croscarmellose Na 2%	0.68 ± 0.01	3.02 ± 0.01	18.81 ± 0.51	1.21 ± 0.33	42.55 ± 1.23
SYLOID 20% avicel:lactose 75:25 PVP 5%	35 ± 0.8	3.04 ± 0.06	22.11 ± 0.39	0.72 ± 0.02	37.19 ± 0.39
SYLOID 20% avicel:lactose 75: 25 PVP 5% crosscarmellouse 2%	3.98 ± 0.06	3.04 ± 0.02	20.33 ± 0.6	0.51 ± 0.18	33.73 ± 0.28

## Discussion

### SYLOID’s Limitations as a Standalone Excipient in Tablet Compression

The results clearly indicate that SYLOID, when used alone, exhibits poor compressibility and mechanical strength. Failure to form tablets at pressures below 221.72 MPa and disintegration upon ejection at this high pressure highlight its unsuitability as a direct compression excipient without additives. This behavior aligns with the fundamental physical characteristics of SYLOID: low bulk density, irregular particle morphology, and brittleness.

Particle fragmentation and reduction in particle size upon compression (from 117.3 to 34.69 μm) confirm brittle fracture rather than plastic deformation. SEM imaging corroborated these findings, revealing surface cracks and detachment of fragments. Such fragmentation disrupts particle cohesion and introduces defects that compromise tablet hardness.

Mesoporous silica carriers, particularly those composed of SiO_2_, are prone to structural squeezing under compression, where pore collapse reduces tablet strength and may lead to premature drug release [28]. Aluminosilicate carriers tend to have better mechanical properties due to their altered network structures and bonding, allowing improved tabletting [29].

Surface chemistry plays a critical role. Silanol groups on SYLOID surface impart hydrophilicity but hinder strong interparticle bonding, limiting cohesive strength in tablets [26]. The lack of plastic deformation capacity results in elastic recovery after compression, promoting capping and lamination [36]. These phenomena explain why SYLOID alone cannot form robust tablets.

Literature suggests that silica-based excipients require plastic diluents [37], often in high proportions (>60% w/w), to improve tabletting by compensating for silica’s brittleness [36]. Without such excipients, silica particles form agglomerates and interparticulate voids [38], resulting in mechanically weak tablets prone to fragmentation and poor handling.

### Enhancing Tabletability Through Diluent Selection

The use of MCC and lactose as diluents demonstrated significant improvements in tablet formation and mechanical properties. MCC’s excellent plastic deformation and binding characteristics reduced tablet porosity and increased mechanical integrity. In contrast, lactose’s brittle fracture mechanism and high solubility contributed to higher porosity and faster disintegration. The interplay between SYLOID and diluents was complex. Increasing SYLOID content increased porosity due to the carrier’s high pore volume and low density, counteracting the densifying effect of MCC. This increased porosity can be advantageous for drug release but presents challenges for mechanical strength and tablet robustness. High SYLOID loading (40%) combined with a low MCC ratio (25:75 avicel:lactose) resulted in tablets with inadequate mechanical integrity, illustrating limits of formulation flexibility. Optimal balance is required to ensure adequate compressibility, disintegration, and mechanical strength.

### Disintegration Mechanisms and Excipient Interactions

Disintegration behaviour was strongly influenced by excipient composition and SYLOID concentration. MCC’s poor swelling and insolubility contribute to longer disintegration times, whereas lactose dissolves readily, facilitating rapid water ingress and tablet breakup.

SYLOID’s physical form influenced water penetration. Unlike smaller MSNs with high surface area and rapid swelling, SYLOID’s larger particle size and limited surface area reduced water absorption and slowed disintegration [15]. The presence of MCC may further impede water penetration by forming dense matrices around SYLOID particles [19, 20]. Achieving a balance between tablet porosity, mechanical strength, and disintegration time requires careful excipient selection and ratio optimisation [18, 21]. Porosity facilitates water ingress and disintegration, but excessive porosity compromises mechanical strength. MCC provides plasticity and strength but slows disintegration; lactose promotes rapid disintegration but may reduce tablet hardness [35].

### Effects of Excipient Composition on Tensile Strength

The observed increase in tensile strength with increasing Avicel content can be attributed to the inherent properties of microcrystalline cellulose (MCC). MCC’s ability to undergo plastic deformation allows its particles to deform and maximise contact areas, facilitating stronger interparticle bonding. This phenomenon is well documented in the literature [10] and aligns with the fundamental principles of tablet compaction.

Hydrogen bonding plays a critical role as well. The hydroxyl groups on the MCC surface enable hydrogen bond formation between adjacent particles, which enhances tablet cohesion and mechanical integrity [31]. This chemical bonding complements the physical deformation and particle rearrangement during compression. The crystalline structure of MCC further supports its superior tabletability. The tightly packed linear chains of glucose in MCC impart mechanical strength and resistance to fragmentation [30]. Additionally, the irregular and elongated shape of MCC particles promotes mechanical interlocking within the tablet matrix, which serves as another layer of strength reinforcement [39]. Thus, the plasticity, hydrogen bonding capability, crystallinity, and particle morphology of MCC collectively explain why increasing Avicel ratios lead to tablets with higher tensile strength.

### Impact of Silica Amount and Particle Size on Tablet Strength

In contrast to MCC, the presence of SYLOID silica negatively affected tablet tensile strength as its concentration increased. This finding is consistent with previous studies reporting that silica excipients can reduce tablet compactability and increase friability due to their rigid structure [40].

The rigid shell of SYLOID is attributed to silanol groups on its surface, which impart limited plastic deformability. This rigidity results in poorer particle bonding during compression compared to the more deformable MCC and lactose particles [25]. The microporous nature of SYLOID may further contribute to its brittleness by restricting particle rearrangement. Particle size is also an important factor. While smaller particle sizes (such as those of mesoporous silica nanoparticles) are known to improve tablet strength by increasing surface area and promoting better bonding [41], the relatively large particle size of SYLOID (∼115 μm) used in this study likely limits these benefits. Larger particles reduce the overall surface area for bonding and decrease the efficiency of particle packing, contributing to the observed decrease in tablet tensile strength as SYLOID content increases.

### Friability Trends and Their Mechanistic Explanation

The friability results complement the tensile strength findings. The highest friability observed in tablets with 40% SYLOID and 50:50 avicel:lactose ratio reflects the lack of plastic deformation and poor interparticle bonding of SYLOID, leading to weak compacts that are more prone to mechanical wear and damage [40]. Friability decreased significantly with increasing Avicel content, which can be explained by the plastic deformation properties and bonding capacity of MCC [42]. As the ratio of MCC increases, the particles deform and bond more effectively, producing harder tablets that resist abrasion and weight loss during friability testing [18]. Additionally, increased SYLOID content reduces the proportion of the plastic diluent mixture (Avicel and lactose), thereby diminishing the overall plastic deformation capacity of the formulation. This reduction limits the ability of the matrix to distribute compressive forces around the rigid silica particles, resulting in less compacted and more friable tablets [18].

### Effects of Excipient Composition on Tablet Thickness

Tablet thickness varied significantly with both the ratio of Avicel to lactose and the amount of SYLOID silica. Increased Avicel content produced thicker tablets, consistent with its plastic deformation behaviour that leads to less volume reduction upon compression [43]. In contrast, lactose monohydrate consists of fine crystals within an amorphous matrix that undergoes plastic deformation but also significant volume reduction upon compression [34]. Therefore, formulations with higher lactose content (25:75 avicel:lactose ratio) yielded thinner tablets. The increase in tablet thickness with higher SYLOID content can be attributed to the rigid and porous structure of the silica, which resists compression. The silanol groups and porous nature of SYLOID limit compressibility, requiring greater applied pressure for compaction and often causing particle fragmentation [24, 25]. This effect results in larger final tablet volumes and increased thickness.

### Optimisation of Tablet Formulation With Binder and Superdisintegrant

Despite attempts to optimise tablet composition, formulations with SYLOID consistently failed to meet pharmacopeial disintegration and friability standards (below 1%) [44]. To address these issues, functional excipients were incorporated. Croscarmellose sodium (CMC-Na), a superdisintegrant, was added at 2% w/w to facilitate rapid tablet disintegration by promoting water uptake and swelling, thereby accelerating tablet breakup [45]. Polyvinylpyrrolidone (PVP), a hydrophilic binder used at 5% w/w, improved mechanical strength by enhancing interparticulate adhesion during compression and reducing friability [46]The binder’s plastic deformation properties increase powder densification and minimise surface cracking and abrasion [46]. The combined use of PVP and CMC-Na yielded tablets that met USP standards, balancing the competing demands of mechanical robustness and rapid disintegration. PVP alone improved friability but extended disintegration time due to increased tablet cohesiveness limiting water penetration. However, the addition of CMC-Na counteracted this effect [47], resulting in rapid disintegration [18]. This synergistic combination provides an optimal formulation for SYLOID-containing tablets.

## Conclusion

This study demonstrates that SYLOID XDP 3150, a mesoporous silica microparticle, can be successfully incorporated into oral tablet formulations when appropriate formulation strategies are applied. Direct compression of SYLOID alone was not feasible due to fragmentation under high compaction pressures, reflecting its poor compressibility and large particle size. However, optimisation of excipient ratios, particularly Avicel PH 102 and lactose, enabled tablets with acceptable mechanical strength and disintegration properties. Tablets with 75% Avicel showed significantly improved tensile strength and reduced friability, while increased SYLOID content enhanced porosity but negatively affected compressibility. Disintegration time was highly dependent on lactose content and was further improved by adding croscarmellose sodium (2%) as a superdisintegrant and polyvinylpyrrolidone (5%) as a binder, which were necessary to meet pharmacopeial requirements. The study underscores the importance of balancing compression pressure, excipient selection, and carrier concentration to address tabletting challenges with mesoporous silica. These findings provide valuable insights into formulating porous silica-based oral dosage forms, supporting their potential as carriers for drug delivery applications requiring high surface area and controlled disintegration. Future work will focus on evaluating drug release profiles from silica-based tablets to assess their suitability as controlled-release delivery systems.

## Summary Table

### What Is Known About This Subject

- Mesoporous silica materials have high surface area and porosity, ideal for drug delivery.
- Their poor compressibility limits use in tablet formulations.

### What This Paper Adds

- Tablet formulation with mesoporous silica is achievable with optimised excipient ratios.
- Superdisintegrants and binders improve tablet strength and disintegration.
- Formulation balance is key to maintaining function and manufacturability.

### Concluding Statement

This work represents an advance in biomedical science by demonstrating how mesoporous silica can be effectively transformed into oral tablets through tailored formulation strategies, enabling their practical use in future drug delivery systems.

## Data Availability

The original contributions presented in the study are included in the article/supplementary material, further inquiries can be directed to the corresponding authors.
